# Development and validation of a health practitioner survey on ocular allergy

**DOI:** 10.1038/s41598-024-60837-6

**Published:** 2024-04-30

**Authors:** Ereeny Mikhail, Mohammadreza Mohebbi, Moneisha Gokhale, Serap Azizoglu, Cenk Suphioglu

**Affiliations:** 1 NeuroAllergy Research Laboratory (NARL), School of Life and Environmental Sciences, Deakin University, Waurn Ponds, Geelong, VIC 3216 Australia; 2 Deakin Optometry, School of Medicine, Deakin University, Waurn Ponds, Geelong, VIC 3216 Australia; 3 Institute for Mental and Physical Health and Clinical Translation (IMPACT), Deakin University, Waurn Ponds, Geelong, VIC 3216 Australia; 4 Biostatistics Unit, Faculty of Health, Deakin University, Geelong, VIC 3216 Australia

**Keywords:** Ocular allergy, Health practitioners, Survey validation, Diagnosis, Disease prevention, Health services, Public health, Epidemiology, Outcomes research, Eye diseases, Immunological disorders, Eye manifestations

## Abstract

Survey studies have played a significant role in understanding the gaps in the knowledge and practices of health practitioners. However, there have been no such survey studies on Ocular Allergy (OA). Thus, the purpose of this study was to develop and validate a survey on OA to better understand the gaps in the diagnostic, treatment, and collaborative care approaches of health practitioners in OA. The survey is titled “Survey on Ocular Allergy for Health Practitioners (SOAHP)”. SOAHP was developed in a five-stage process. First, item extraction via the use of a literature review, second, face and content validity, third, a pilot study, fourth, test–retest reliability, and fifth, finalisation of the survey. 65 items under 6 domains were initially generated in the item extraction phase. Content validity was conducted on 15 experts in the field. This was conducted twice to reach consensus whereby items and domains were added, edited, kept, or removed, resulting in 50 items under 7 domains. The pilot study was conducted on 15 participants from the five relevant health practitioner fields (Allergists/Immunologists, General Practitioners (GPs), Ophthalmologists, Optometrists and Pharmacists). This altered the survey further to 40 items under 7 domains. Test–retest reliability was conducted on 25 participants from the five health practitioner fields. Reliability was moderate to almost perfect for most (97%) investigated items. The finalised survey was 40 items under 7 domains. SOAHP is the first survey created to assess diagnostic, treatment and collaborative care approaches of Allergists/Immunologists, GPs, Ophthalmologists, Optometrists and Pharmacists on OA. SOAHP will be a useful tool in clinical research on OA.

## Introduction

Ocular Allergy (OA) refers to a spectrum of ocular allergic diseases of different severities and underlying pathophysiology^1^. These diseases lead to debilitating effects on the quality of life (QoL) of affected individuals^2–6^. OA may also lead to sequelae of other ocular diseases including dry eye^7^ and keratoconus^8^, which lead to further effects on QoL^9,10^ and severe visual impairment^11^. Further to this, the prevalence of allergy has been increasing worldwide^12^ and specifically, in Australia^13^. In fact, in 2016, the world’s most catastrophic thunderstorm asthma event occurred in Melbourne, Australia, due to the pollen of ryegrass (*Lolium perenne*). This resulted in 10 deaths and 3365 excess respiratory-related cases presenting to public hospital emergency departments, which was a 672% increase than the 3-year average of 501^13^. With known serious ocular effects and the increasing prevalence of allergy^12,13^, there needs to be a more effective, efficient and unified, diagnostic, treatment and collaborative care approach to OA.

OA is generally diagnosed and managed by Allergists/Immunologists, General Practitioners (GPs), Ophthalmologists, Optometrists, and Pharmacists in Australia^14–16^. However, the literature has shown disparities in these health practitioners’ diagnosis and management of the disease^17–19^. Yet the exact gaps in the knowledge and practices of that lead to these disparities are unknown. Thus, it is essential to understand current gaps in health practitioner diagnostic, treatment, and collaborative care approaches to OA. Through this, there will be less clogging of the healthcare system, a reduced economic burden, and less complications of OA^20–22^.

Previous literature has shown that survey studies have aided in understanding health practitioner gaps in knowledge and practices surrounding a health condition^23–26^. Through this, the disparities amongst health practitioners were alleviated and improved practices employed^23–26^. However, this has been an overlooked concept in OA. At present there are no surveys that have been validated to assess all aspects on OA. Therefore, the role of a health practitioner survey on OA is significant to address this health issue. Thus, the purpose of this paper is to develop such a survey to assess health practitioner diagnostic, treatment, and collaborative care approaches to OA.

## Methods

The development and validation of the Survey on Ocular Allergy for Health Practitioners (SOAHP) followed a five-step method. This was established through consideration of the papers by Rodrigues et al*.*^27^, Hoffman et al*.*^26^, and Howell et al*.*^28^, the guidelines by Boateng et al*.*^29^, and Tsang et al*.*^30^, and the reliability and validity methods discussed by Mikhail et al*.*^31^. The five-step method involved; (1) item extraction, (2) face and content validity, (3) pilot study, (4) test–retest reliability, and (5) finalisation. The details of these methods are explained in detail below. Participants were recruited via publicly available email addresses. This study adhered to the tenets of the Declaration of Helsinki and human ethics was approved by Deakin University (reference number: SEBE-2020-68-MOD01). All methods were performed in accordance with the ethics approval and consent to participate was ensured through a plain language statement and consent form for participants to sign.

### Step one: item extraction

Items and domains were created by the researchers in this study, via the use of a literature review. This is an established method in the current literature^4–6,32–35^. This covered the three aspects of diagnosis, treatment, and collaborative care^27^.

The terminology referring to OA used in the literature search included ‘ocular allergy,’ ‘allergic rhinoconjunctivitis,’ ‘allergic conjunctivitis,’ ‘allergic eye disease,’ ‘acute allergic conjunctivitis’, ‘seasonal allergic conjunctivitis,’ ‘perennial allergic conjunctivitis,’ ‘vernal keratoconjunctivitis,’ ‘atopic keratoconjunctivitis,’ ‘giant papillary conjunctivitis,’ and ‘contact blepharoconjunctivitis.’ A general search using these terms was made which then stemmed into further searches as detailed below.

Following the initial general search on OA, QoL appeared to be a significant topic in OA^2–6,18,19,33–50^. Therefore, a deeper search was conducted on this topic using the terms, ‘quality of life,’ ‘questionnaires,’ and ‘surveys.’ These keywords were used alongside the terms referring to OA mentioned above. 7 items were extracted, which included 2 items on awareness of QoL questionnaires, 3 items on implementation of QoL questionnaires, and 2 items covering reasons of use of QoL questionnaires. These formed the *OA QoL Questionnaires* domain.

Another topic of significance, in the initial search on OA, was patient history^1,31,40,51–67^. Thus, another literature search was conducted on this topic using the following terms; ‘history taking,’ ‘symptoms,’ and ‘signs.’ These keywords were used in multiple different arrangements alongside the terms referring to OA mentioned. 6 items were extracted, whereby 2 assessed awareness of types of OA, 1 assessed hallmark symptom of OA, 1 assessed all symptoms on OA, and 2 were targeted at eye rubbing. These formed the *OA History Questions* domain.

Likewise, in the initial search of OA, consideration of differential diagnosis appeared to be another significant topic^58,68–81^. Thus, a further literature search was conducted using the terms ‘red eye,’ and ‘differential diagnosis.’ These keywords were combined with the OA terms mentioned previously. 1 item was extracted, which covered all differential diagnosis on OA. These formed the *Differential Diagnosis of OA* domain.

Further, diagnostic tools in OA was another significant topic found in the initial literature search on OA^56,68,82–99^. Thus, an in-depth literature search included the terms ‘diagnostic methods,’ and ‘diagnosis.’ These keywords were arranged alongside the terms used for OA above. 2 items were extracted, which covered all diagnostic methods used in OA and referral pathways for diagnosis of OA. These formed the *Diagnostic Methods in OA* domain.

Moreover, management of OA was another significant topic found in the initial literature search^8,9,52,54,72,82,100–113^. Thus, a deeper literature search was conducted using terms including ‘management,’ and ‘treatment’. These keywords were combined with those referring to OA above. 45 items were extracted, which included 2 items covering all treatments in OA that was then broken down into 4 items on prevention strategies, 5 items on symptom and cosmetic remedies, 17 items on topical drops (including both allergy specific and anti-inflammatory eye drops), 6 items on systemic treatments, 8 items on referral pathways for management, and 3 items on knowledge of managements (which covered immunology of OA). These formed the *Management Methods in OA* domain.

Finally, collaborative care was another topic identified in the initial literature search on OA^14–16,40,114–124^. Thus, a further search was made using the terms ‘collaborative care,’ ‘interdisciplinary collaborations,’ and ‘health practitioners.’ 3 items were extracted. These formed the *Collaborative Care in OA* domain.

Additionally, it should be mentioned that 1 item allowed for additional information to be provided. Thus, the only headings in the literature review, which were not covered in SOAHP, were Epidemiology and Systemic Allergy, as these were found to be irrelevant to clinical practice, or difficult to be adapted to the five types of health practitioners, respectively.

The form of the items (i.e. single selection responses, multiple selection responses or open ended questions) was selected through researcher deliberation. Items were altered in the validation methods.

### Step two: face and content validity

It is generally recommended that 2 to 20 experts are involved in face and content validity^125^. The experts (n = 15) involved in this process included 1 Allergist/Immunologist, 1 Ophthalmologist, 1 General Paediatrician who is a researcher in Allergy, Asthma and Immunology, 3 Optometrists, 3 GPs, 3 Pharmacists, and 3 OA Researchers. Experts were selected based on the following guidelines: (a) involved in the care of OA patients, (b) is one of the health practitioners the final survey will be administered to, and/or (c) involved in allergy, asthma and immunology research.

Face validity was first conducted to assess if each item was suitable to the purpose of the topic of OA. Following this, content validity was conducted via the use of the modified Delphi technique^126^. This technique involves pre-meditated answers to each item in the survey whereby experts in the field assess the items and pre-meditated answers, until consensus is reached.

Experts assessed the relevance, essentiality, and clarity (according to a well-established scale) of each item in the domains^27^. The established Likert scales for relevance, essentiality, and clarity were selected from the Rodrigues et al.^27^ paper. The scale for relevance was a 4-point Likert scale, which was *1* = *not relevant, 2* = *somewhat relevant, 3* = *quite relevant* and *4* = *very relevant*, whereby 1 and 2 were considered content-not-relevant and 3 and 4 were considered content-relevant^27^. The scale for essentiality was also on a 3-point Likert scale whereby *1* = *not essential, 2* = *useful, but not essential* and *3* = *essential*, whereby 3 was considered essential^27^. Finally, the scale for clarity was a 3-point Likert scale where *1* = *not clear*, *2* = *item needs some revision* and *3* = *very clear*^27^. This was conducted on Qualtrics, Provo, UT^126^. Finally, an open section for suggestions was provided to ensure nothing important surrounding the topic had been missed and to make appropriate amendments to the items.

Data was analysed through Content Validity Index (CVI) using item-CVI (I-CVI) for Relevance, Content Validity Ratio (CVR) for Essentiality, and Averaging Scores for Clarity^27^. I-CVI was calculated for each item as the number of experts rating the item as 3 or 4 (“*quite relevant*” and “*very relevant*”), divided by the total number of experts^127^. Values range from 0 to 1, where I-CVI > 0.79 means the item is relevant, between 0.70 and 0.79 means the item needs some revisions and below 0.70 means the item is removed^27^. CVR is calculated using the formula CVR = (Ne − N/2)/(N/2), where Ne is the number of panellist’s indicating the item as 3 (“*essential*”) and N is the total number of panellist’s^27,127,128^. Values range from 1 to − 1, and based on the numerical values in Lawshe’s table for n = 15 experts, CVR = 0.49 was the minimum value for an item to be considered essential^128^. For clarity, the scoring by each expert was averaged for each item and if comments were provided, the item was clarified. Finally, any comments regarding adding questions, editing questions, and removing questions were implemented, if deemed justifiable (e.g. if an item was suggested to be removed, but found to be important to the topic of OA and to be the only item covering this topic, then this was kept but edited as per comments provided).

### Step three: pilot study

SOAHP was then administered to 15 participants^129^ (3 from each of the 5 specialities) who were not the same as those in the content validity phase, via Qualtrics, Provo, UT. This included 3 Allergists/Immunologists, 3 GPs, 3 Ophthalmologists, 3 Optometrists and 3 Pharmacists. The health practitioners needed to be Australian Health Practitioner Regulation Agency (AHPRA) registered and practicing in Australia. Health practitioners who were in the five health fields but were not fully qualified (i.e. students, interns, residents, registrars, and/or in training) were excluded and not allowed to participate in the project. Any other health practitioner not mentioned (e.g. Dermatologists) was also excluded. This was to ensure the pilot study simulated the purpose of SOAHP, which is to assess these five, fully qualified health practitioners’ knowledge and practices on OA.

The respondents were examined on how they ‘comprehended, interpreted and answered’ the survey questions^27^. This means the respondents were assessed to see if they all had the same understanding of the questions. Thus, the participants were asked if there were any difficulties following the wording of the questions or other problems that may lead to response error or bias. Additionally, data on time taken to complete the survey was also collected via Qualtrics, Provo, UT. It is important to note that data collected in the pilot study was not for the purpose of having a representative sample of health practitioner knowledge and practices but was collected to ensure the survey was piloted before wider administration and adjusted, if required.

### Step four: test–retest reliability

SOAHP was administered via Qualtrics, Provo, UT to 25 participants, who were not the same as those from the content validity and pilot study phases^130^, to assess test–retest reliability. The five health practitioner groups were included: 2 Allergists/Immunologists, 5 GPs, 3 Ophthalmologists, 8 Optometrists and 3 Pharmacists. The same inclusion and exclusion criteria used in the pilot study was implemented in this step. An equal representation of each health practitioner was preferred but not necessary as the purpose was to assess reliability of the survey. These participants completed the survey twice at different times (in a 1–2 week time frame)^28^ to assess if the same responses were selected on both occasions.

Analysis was conducted on an item-by-item basis using percentage change, percentage agreement, intraclass correlation coefficient (ICC), and percent of agreement for dichotomised index. Percentage change was employed for questions with a high number of response selections. The average percentage change and its standard deviation were calculated for all the responses. An average of < 18 is considered almost perfect, 19–36 is strong, 37–65 is moderate, 66–85 is weak, 86–96 is minimal and > 96 is none^131^. Percentage agreement was used for questions with yes/no responses and correct/wrong responses. This was evaluated by assessing if the same item was chosen in the first and second completion of the survey. The expected score should be greater than 0.90 for almost perfect agreement, 0.80–0.90 for strong agreement, 0.60–0.79 for moderate agreement, 0.40–0.59 for weak agreement, 0.21–0.39 for minimal agreement and 0–0.20 for no agreement^131^. 95% confidence intervals were also calculated and reported. Furthermore, ICC was calculated for ordinal scales (e.g. never, rarely, sometimes, frequently and always). ICC > 0.9 is considered excellent, 0.75–0.9 is considered good, 0.5–0.75 is considered moderate and ICC < 0.5 is considered poor reliability^132^. For questions with nominal responses, agreement was measured by creating a dichotomised indicator that measured whether the same responses were selected the first and second time. This stratified the test–retest comparisons into full agreement (i.e. the same responses selected the first and second time), partial agreement (i.e. some of those selected the first time were also selected the second time) and complete disagreement (i.e. none of those selected the first time were selected the second time). The above cut-offs for percentage agreement was also used for the dichotomised index^131^.

### Step five: finalisation of survey

SOAHP was then finalised ensuring the items and domains were clear, relevant, essential, accurate and consistent. It is essential to note that this survey was a smart survey, whereby certain items only appear if specific answers are selected. Not all items are assessed on each participant. This was a feature applied in Qualtrics, Provo, UT. The finalisation of SOAHP is purposed for wider administration to the five groups of health practitioners, who are AHPRA registered, fully qualified, and practicing in Australia.

### Ethics approval and consent to participate

This study received Deakin University ethics approval (reference number: SEBE-2020-68-MOD01). Consent to participate was ensured through a plain language statement and consent form for participants to sign.

## Results

### Step one: item extraction

**Figure 1:**
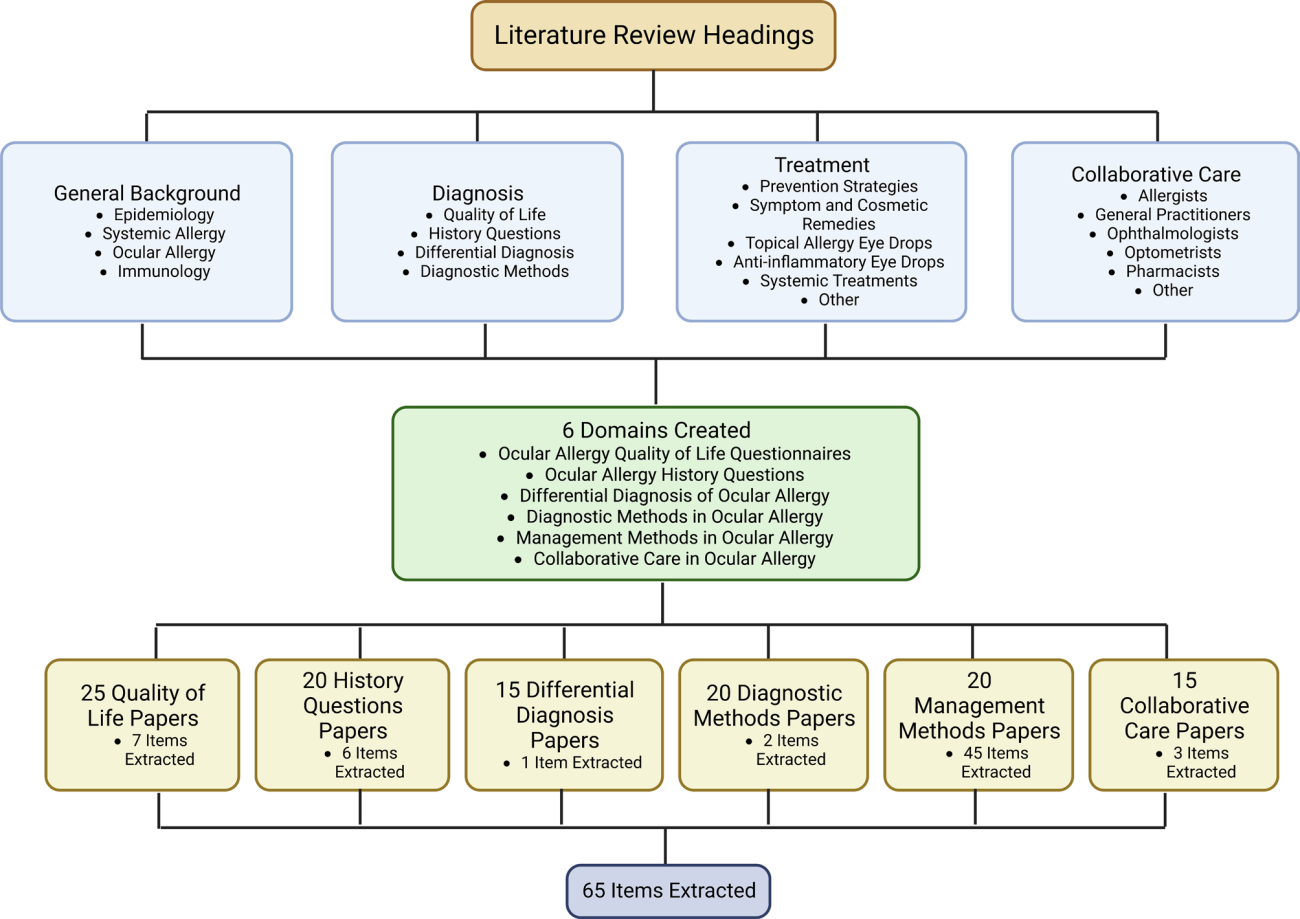
Flow chart of the item extraction process.

As aforementioned, the SOAHP items and domains were extracted through researcher deliberation via a literature review. The literature review covered the headings of general background, diagnosis, treatment and collaborative care, whereby appropriate subheadings were placed under these primary headings. Through the analysis of approximately 115 papers, 65 items were isolated, as they were considered significant to the topic of OA and placed under 6 domains including: OA QoL Questionnaires, OA History Questions, Differential Diagnosis of OA, Diagnostic Methods in OA, Management Methods in OA, and Collaborative Care in OA. This phase was conducted over a 6-month period. This process is demonstrated in Fig. 1.

### Step two: face and content validity

Fifteen experts participated in the face and content validity process, with a recruitment response rate of 40%. Table 1 describes the participant characteristics. There was an almost equal split between males and females, with a mean age of 44.5 ± 12.6 years and mean years of practice of 18.5 ± 13.1. Participants were heavily based in the Australian states of New South Wales and Victoria with 1 Participant from Queensland. Participants worked under different modalities and places of practice. Data was collected over a period of 11 weeks; 6 weeks for Round 1 and 5 weeks for Round 2. There was no participant dropout between Rounds 1 and 2.

**Table 1:** Demographic characteristics of participants in content validity (n = 15), pilot study (n = 15), and test–retest reliability (n = 25).

Characteristic	Content validity, n (%)	Pilot study, n (%)	Test–retest reliability, n (%)
Sex			
Male	7 (46.7)	6 (40)	13 (52)
Female	8 (53.3)	9 (60)	12 (48)
Age (Years)			
Range	24–61	25–60	24–70
Mean	44.5	42.5	38.4
Years of practice			
Range	2–38	1–26	2–48
Mean	18.5	13.7	12.4
State			
Australian Capital Territory	0 (0)	0 (0)	0 (0)
New South Wales	6 (40)	10 (66.7)	16 (64)
Northern Territory	0 (0)	1 (6.7)	0 (0)
Queensland	1 (6.7)	0 (0)	2 (8)
South Australia	0 (0)	0 (0)	0 (0)
Tasmania	0 (0)	0 (0)	0 (0)
Victoria	8 (53.3)	4 (26.6)	3 (12)
Western Australia	0 (0)	0 (0)	4 (16)
Modality of practice			
Full time	8 (53.3)	9 (60)	15 (60)
Part time	4 (26.7)	4 (26.7)	6 (24)
Locum/Casual	3 (20)	2 (13.3)	4 (16)
Other	0 (0)	0 (0)	0 (0)
Place of practice			
Group practice	7 (46.7)	9 (60)	12 (48)
Hospital	5 (33.3)	2 (13.3)	7 (28)
Solo/Individual practice	3 (20)	3 (20)	3 (12)
Community health centre	0 (0)	1 (6.7)	1 (4)
Other	0 (0)	0 (0)	2 (8)

**Figure 2:**
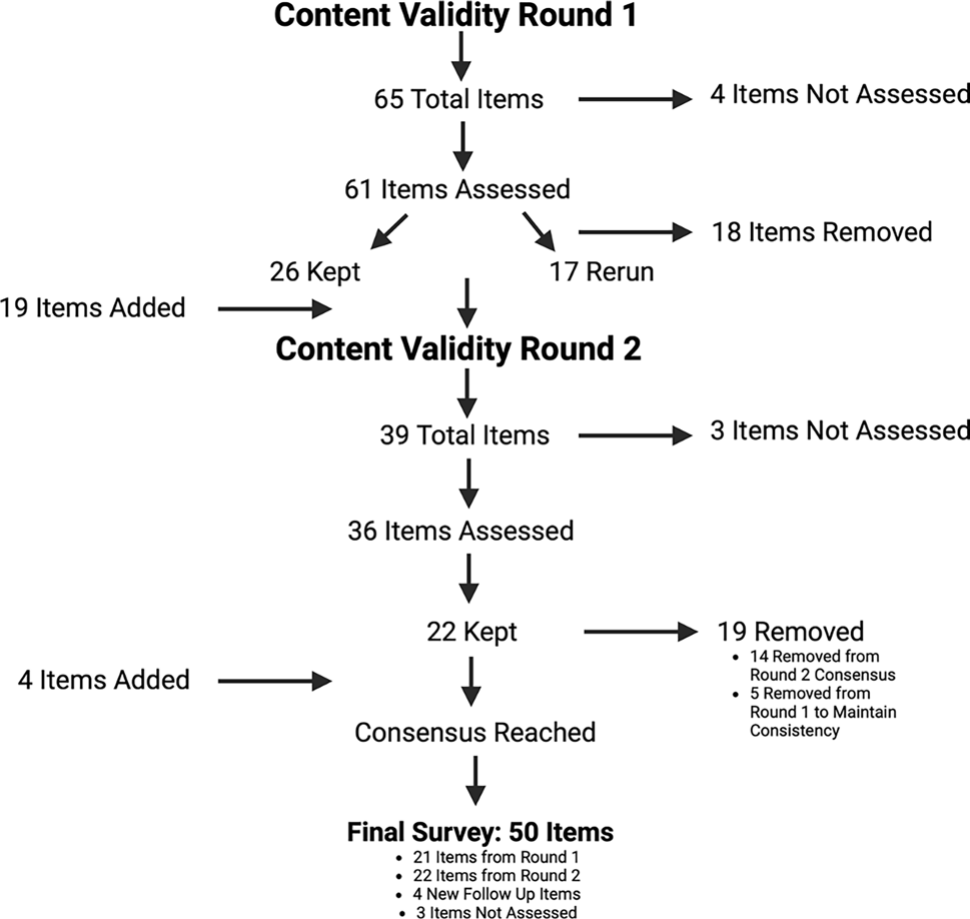
Flow chart of the content validity items that were kept, removed, rerun and added.

There were 65 total items in the first round of content validity. The results are summarised in Table 2. Face validity was deemed appropriate for all items. For content validity, 61 items were assessed, meaning 4 items were not assessed. Items not assessed were follow-up questions and thus, to reduce the burden of the survey on participants, they were not included in the content validity phases. This means that if the item relating to this follow-up question was removed then the follow-up question would also be removed. Based on relevance and/or essentiality, 17 items (27.9%) did not reach consensus and thus, were required to be re-run in the second round. 44 items reached consensus, whereby 26 items (42.6%) were kept in the survey and 18 items (29.5%) were removed from the survey. This process is demonstrated in Fig. 2. Overall, clarity of all items was 2.79 out of 3, revealing that the survey was clear.

**Table 2:** Content validity round 1 and 2 results for relevance, essentiality, and clarity.

	Round 1	Round 2
	Number of items (%)	Number of items (%)
Relevance		
I-CVI 1	6/61 (9.8)	1/23 (4.3)
I-CVI 0.93	8/61 (13.1)	5/23 (21.8)
I-CVI 0.86	15/61 (24.6)	8/23 (34.8)
I-CVI 0.8	16/61 (26.2)	4/23 (17.4)
I-CVI 0.73	7/61 (11.5)	3/23 (13.1)
I-CVI 0.66	5/61 (8.2)	0/23 (0)
I-CVI 0.6	3/61 (4.9)	1/23 (4.3)
I-CVI 0.53	1/61 (1.7)	1/23 (4.3)
Essentiality		
CVR 1	1/61 (1.7)	0/35 (0)
CVR 0.86	3/61 (4.9)	0/35 (0)
CVR 0.73	9/61 (14.8)	1/35 (2.9)
CVR 0.6	17/61 (27.8)	8/35 (22.8)
CVR 0.46	7/61 (11.5)	8/35 (22.8)
CVR 0.3	8/61 (13.1)	3/35 (8.6)
CVR 0.2	2/61 (3.3)	3/35 (8.6)
CVR 0.06	3/61 (4.9)	4/35 (11.4)
CVR − 0.06	6/61 (9.8)	3/35 (8.6)
CVR − 0.2	3/61 (4.9)	2/35 (5.7)
CVR − 0.3	2/61 (3.3)	2/35 (5.7)
CVR − 0.46	0/61 (0)	1/35 (2.9)
Clarity		
Clarity 3	4/61 (6.5)	3/19 (15.7)
Clarity 2.93	13/61 (21.3)	3/19 (15.7)
Clarity 2.86	9/61 (14.8)	4/19 (21.1)
Clarity 2.8	9/61 (14.8)	1/19 (5.3)
Clarity 2.73	15/61 (24.6)	6/19 (31.6)
Clarity 2.67	4/61 (6.5)	1/19 (5.3)
Clarity 2.6	3/61 (4.9)	0/19 (0)
Clarity 2.53	3/61 (4.9)	1/19 (5.3)
Clarity 2.33	1/61 (1.7)	0/19 (0)

For the second round of content validity, there were 39 items. 3 items were those that were not assessed. Thus, 36 items were assessed; 17 were re-runs from the previous round and 19 were new questions suggested by experts. The results are summarised in Table 2. Consensus was reached whereby 22 items (61%) were kept and 14 (39%) were removed. This process is demonstrated in Fig. 2. However, it is significant to note that 5 items that were initially accepted in content validity round 1 were removed in content validity round 2, to maintain consistency of questions. These items were regarding prescribing patterns, however, as some items surrounding prescribing patterns were classed as not relevant and not essential, and thus, removed, then these items were also removed to ensure the survey was consistent. Furthermore, 4 new items were added, based on expert feedback and further deliberation. Overall, clarity for the 19 new items was 2.82 out of 3, again revealing the items were clear.

The final survey following content validity was 50 items under 7 domains. These domains were Red Eye Case Scenario, QoL of OA Patients, OA History Questions, Diagnostic Methods in OA, Management Methods in OA, Knowledge on OA, and Collaborative Care in OA. The addition and removal of domains was based on the alteration of the items in the content validity process due to expert feedback. This was deliberated on by the researchers in this study.

### Step three: pilot study

Fifteen participants were involved in the pilot study phase with a recruitment response rate of 25%. Participant characteristics are described in Table 1. There were 40% males and 60% females involved, with a mean age of 42.5 ± 11.6 years and mean years of practice being 13.7 ± 9.0. Participants were again heavily based in New South Wales, and Victoria with 1 participant from the Northern Territory. Like the content validity phase, participants worked under different modalities and places of practice.

The pilot study data was collected over a period of 4 weeks. The average completion time was 37.20 ± 17.4 min. There was an exclusion of one participant's time who was an outlier with an 87-min difference between this participant and the participant with the longest completion time. Participants provided comments, which resulted in the removal of 12 items and the addition of 2 items. The 12 items removed were regarding the management domain. They were all similar items assessing which management is applied to the different types of OA. However, it was found that these items required more context, as managements were applied depending on the severity of the OA, rather than the type, which was a notion expressed by most participants. Overall, SOAHP was clear to all participants. The pilot study reduced the survey to 40 items under the same 7 domains identified in the content validity phase. Thus, it was expected SOAHP would have a shorter completion time during the wider survey distribution.

### Step four: test–retest reliability

SOAHP was administered to twenty-five participants for the test–retest reliability phase with a recruitment response rate of 57%. Participant characteristics are described in Table 1. There was almost an equal split between males and females, with a mean age of 38.4 ± 13.6 years, and mean years of practice being 12.4 ± 12.7. Participants were again heavily based in New South Wales with some participants from Victoria, Queensland, and Western Australia. Like the other phases, participants worked under different modalities and places of practice.

Data was collected over a period of 11 weeks and there was an average time of 10.92 ± 4.6 days between the first and second responses. The 40 items under the 7 domains identified in the pilot study were in the survey but only 39 items were analysed as the final item was an open-ended question regarding additional information. For the 39 items analysed, 14 were analysed by percentage change, 13 were analysed by percentage agreement, 4 were analysed using ICC, 6 were analysed using percent of agreement for dichotomised index and 2 were not analysed due to having only one participant response. A summary of the results are shown in Table 3. Overall, test–retest reliability was moderate to almost perfect for 97% of items assessed.

**Table 3:** Test–retest reliability results for percentage change, percentage agreement, ICC, and dichotomised index.

	Number of items (%)
Percentage change	
< 18 (Almost perfect)	14/14 (100)
19–36 (Strong)	0/14 (0)
37–65 (Moderate)	0/14 (0)
66–85 (Weak)	0/14 (0)
86–96 (Minimal)	0/14 (0)
> 96 (None)	0/14 (0)
Percentage agreement	
> 0.90 (Almost perfect)	3/13 (23.1)
0.80–0.90 (Strong)	6/13 (46.2)
0.60–0.79 (Moderate)	3/13 (23.1)
0.40–0.59 (Weak)	0/13 (0)
0.21–0.39 (Minimal)	1/13 (7.6)
0–0.20 (None)	0/13 (0)
ICC	
> 0.9 (Excellent)	3/4 (75)
0.75–0.9 (Good)	1/4 (25)
0.5–0.75 (Moderate)	0/4 (0)
< 0.5 (Poor)	0/4 (0)
Dichotomised index	
> 90% (Almost perfect)	1/6 (16.7)
80–90% (Strong)	3/6 (50)
60–79% (Moderate)	2/6 (33.3)
40–59% (Weak)	0/6 (0)
21–39% (Minimal)	0/6 (0)
0–20% (None)	0/6 (0)
Not assessed	2/2 (100)

### Step five: finalisation of survey

The survey remained unchanged following the pilot study phase, which was an essential criterion to ensure test–retest reliability was accurate. The finalised survey included 40 items under the 7 domains, as described previously. This included 7 items under the *Red Eye Case Scenario* domain, which covered several topics including 1 item on history questions on OA, 1 item on differential diagnosis of OA, 1 item to diagnose the case scenario, 2 items on management methods in OA, and 2 items on referral pathways. Furthermore, the *QoL of OA Patients* domain had 3 items, which covered implementation and awareness of QoL in OA. The *OA History Questions* domain had 5 items, including 2 items on awareness of types of OA, 1 item on the hallmark symptom of OA, 1 item on all symptoms of OA, and 1 item on eye rubbing. Moreover, the *Diagnostic Methods in OA* domain included 2 items, on all diagnostic methods used in OA and referral pathways for diagnosis of OA. The *Management Methods in OA* domain had 9 items, with 2 items that broadly covered all types of management methods in OA, then 5 items which looked for the specific management methods in OA, and 2 on referral pathways for management of OA. Further, the *Knowledge on OA* domain had 9 items, with 3 items on immunology of OA, 3 items were on side effects and/or precautions of management methods, and 3 items on other considerations in the management of OA. Finally, the *Collaborative Care in OA* domain had 4 items to gauge on points of view and referral pathways in OA. An additional item was provided for comments.

The breakdown of results for Step 2–4 can be seen in Supplementary Material 1.

## Discussion

SOAHP is the first validated survey, which assesses Allergists/Immunologists, GPs, Ophthalmologists, Optometrists and Pharmacists knowledge and practices on OA. SOAHP aimed to encompass all domains on OA to better understand these health practitioner diagnostic, treatment and collaborative care approaches to OA. There are 7 domains, excluding demographics: Red Eye Case Scenario, QoL of OA Patients, OA History Questions, Diagnostic Methods in OA, Management Methods in OA, Knowledge on OA, and Collaborative Care in OA. Although the survey was developed with the Australian healthcare model and scopes of practices in mind, this survey can be easily adapted and administered globally, but warrants validation in each country depending on the healthcare model of that country, and scope of practice of each health practitioner.

Although initially not included, the expert comments and further deliberation of researchers found that a case scenario domain was required. The implementation of a case scenario domain was further motivated by Ferreira^133^, to assess current approaches of health practitioners in real-life case scenarios. This domain, although helpful, can be omitted from the survey without affecting the validation, to allow for a shorter survey. However, the inclusion of a case scenario in SOAHP allows to gauge deeper understanding of gaps in knowledge and practices on OA, as it simulates a clinical environment^133^ and covers all domains on OA. Further to this, it was able to encapsulate other domains such as the initial differential diagnosis domain which only had 1 item. Thus, this domain was removed as it was covered in the case scenario.

The quality-of-life domain assesses awareness and implementation surrounding currently available questionnaires^31^, which has not been assessed previously^120,134^.

The history questions domain, for the first time, assesses the awareness of health practitioners of the different presentations of OA^120^. A prominent survey conducted in USA (2014) on OA for health practitioners (n = 500), was the AIRS survey^120^, which lacked specific history questions on OA. Instead, vague questions, (e.g. main symptom that resulted in patient attendance) were employed^135^. Practitioners stated that ‘itchy eyes’ (62%), being the hallmark symptom of OA, was the most common reason patients sought care^135^. However, no follow up questions were examined, such as eye rubbing, which is essential as this may lead to severe negative effects including ocular conditions such as keratoconus, which leads to progressive visual loss. The other survey study conducted in Italy (2015) on health practitioners (n = 200) was on allergic rhinitis, which revealed that GPs diagnosed the majority of allergic rhinitis cases^134^. However, ocular symptoms noted in the survey did not include ‘itchy eyes’ as one of the accompanying symptoms^134^. Instead, tearing, redness and conjunctivitis were noted. It is alarming that the hallmark symptom of OA (i.e. ‘itchy eyes’) was not queried. Therefore, targeted history taking items were implemented in SOAHP.

The diagnostic methods in OA domain aimed to encompass the variety of tools used by the five different health practitioners in diagnosing OA, which will likely deepen the understanding of the scopes of practice. This is significant as previous literature has revealed disparities in diagnostic methods of different health practitioners in allergic rhinitis^120^. The AIRS study found that most of those with allergic rhinoconjunctivitis were diagnosed by GPs (46% for age 18 + and 22% for < 18), rather than allergists/immunologists (17% for 18 + and 19% for < 18). However, only 37.4% of GPs employed an allergy test (i.e. skin prick or blood test), compared with 94.9% of allergists/immunologists^120^. Although, disparities were identified in practices, this did not aid in creating an appropriate model for scopes of practices to aid in a more efficient diagnosis, management, and collaborative care of OA patients. Since the AIRS study only included few diagnostic methods, SOAHP implemented an item that included *all* diagnostic methods of health practitioners involved in OA to better understand current gaps and therefore, scopes of practice. Through this, an appropriate model of care can be created.

The management methods in OA domain focused on practices of health practitioners in OA, whilst the knowledge on OA domain implemented items with correct and wrong answers to assess knowledge. This has not been gauged previously^105,120^. Treatment questions in the AIRS study were also not comprehensive for OA (i.e. studies only mentioned recommending immunotherapy referrals to aid in decreasing ocular symptoms). Although significant, other OA-specific treatments should have been questioned^105^. Likewise, in the Canonica et al*.* (2015) study, ocular treatments were not assessed as a targeted approach to these patients^134^. Thus, more targeted questions on ocular specific treatments were applied in SOAHP (e.g. topical treatments) in order to create a more effective treatment system surrounding OA, whereby scopes of practice can be further defined.

Finally, collaborative care in OA items were implemented throughout the survey, but also had a specific domain aimed at understanding the communication between health practitioners in OA, which is lacking in the current literature^120,134^. Current studies merely create collaborative care models without any profound evidence^136^. Thus, the need for an evidence based collaborative care model is required.

The survey had overall high content validity: I-CVI ranging from 0.73 to 1.00, average CVR 0.53, and average item clarity of 2.80. Moreover, the pilot study provided information on the participant’s understanding of the survey, thereby permitting necessary edits. Finally, the test–retest reliability phase allows researchers to ensure that the survey is reliable. One of the unforeseen effects of validation, which to our knowledge has not been mentioned in previous literature, is the power of the validation process (e.g. participant feedback), to result in the critical analysis of the items and domains in the survey. Through this, the addition, removal and/or re-establishing of items and domains is instigated. The final version of SOAHP is available as Supplementary Material 2.

## Conclusions

SOAHP was formed as a response to current disparities in health practitioner approaches to OA. Additionally, with the aforementioned, effects of OA and the increasing prevalence, there needs to be a more unified approach. SOAHP was validated using appropriate methods to ensure it captures gaps in the knowledge and practitioners of relevant health care practitioners including Allergists/Immunologists, GPs, Ophthalmologists, Optometrists and Pharmacists. This will add fundamental knowledge to the current literature, whereby improved education can be implemented.

### Supplementary Information

Supplementary Information 1.

Supplementary Information 2.

## Data Availability

Data breakdown and final survey available in Supplementary Materials 1 and 2, respectively.
