# Supplementary material for: Development and validation of a health practitioner survey on ocular allergy
Source: Sci Rep. 2024 Apr 30;14:9932. doi: 10.1038/s41598-024-60837-6 (PMC11061311; doi:10.1038/s41598-024-60837-6)
Supplement: Supplementary file 1 — Supplementary Information 1. [file 41598_2024_60837_MOESM1_ESM.docx]

| Content Validity Round 1 | | | | |
| --- | --- | --- | --- | --- |
| ITEM | **RELEVANCE** | **ESSENTIALITY** | **CLARITY** | **DECISION** |
| 1a. Are you AWARE of any of the following ocular allergy focused quality of life questionnaires? | 0.6 | -0.2 | 2.93 | This item was rated as not relevant and not essential. Therefore, this item was removed. |
| 1b. Choose which questionnaire(s) you are AWARE of. | - | - | - | This item was not run as this was a follow-up question. This was automatically removed as 1a was removed. |
| 2a. Do you USE any of the following ocular allergy focused quality of life questionnaires in your practice? | 0.8 | -0.06 | 3 | This item was rated as relevant but not essential. This item was re-run in the second round of content validity. |
| 2b. Choose which questionnaire(s) you USE. | - | - | - | This item was not run as this was a follow-up question. This item was also not re-run. |
| 2c. Choose the reason why you DO NOT USE these questionnaires. | 0.86 | 0.3 | 2.67 | This item was rated as relevant but not essential. This item was re-run in the second round of content validity. |
| 3a. For what purpose do you USE quality of life questionnaires and/or ask quality of life questions. | 0.8 | -0.06 | 2.8 | This item was rated as relevant but not essential. This item was re-run in the second round of content validity. |
| 3b. When using these questionnaire(s) for the MANGEMENT of patients, do you use it on patients with treatment, without treatment or both? | 0.66 | -0.3 | 2.73 | This item was rated as not relevant and not essential. Therefore, this item was removed. |
| 4a. Which types of ocular allergy are you AWARE of? | 0.86 | 0.6 | 2.93 | This item was rated as relevant and essential. Therefore, this item was kept. |
| 4b. Choose the reason(s) why you selected “None of the Above/Unsure”. | 0.8 | 0.2 | 2.86 | This item was rated as relevant but not essential. This item was re-run in the second round of content validity. |
| 5. Which of the following is the hallmark symptom of ocular allergy? | 1 | 0.6 | 2.8 | This item was rated as relevant and essential. Therefore, this item was kept. |
| 6. Which symptom(s) do you ask your patients about when diagnosing ocular allergy? | 1 | 0.73 | 3 | This item was rated as relevant and essential. Therefore, this item was kept. |
| 7a. When conducting your ocular allergy history taking, do you differentiate between the terms ‘itchy eye’ and ‘eye rubbing’? | 0.66 | -0.06 | 2.6 | This item was rated as not relevant and not essential. This item was removed as it created some confusion. |
| 7b. How often do you ask the patient if they rub their eyes? | 0.86 | -0.06 | 2.67 | This item was rated as relevant but not essential. This item was re-run in the second round of content validity. |
| 8. Which of these do you consider in your differential diagnosis of ocular allergy? | 0.86 | 0.46 | 2.8 | This item was rated as relevant but not essential. This item was re-run in the second round of content validity. |
| 9a. When diagnosing ocular allergy, which diagnostic method(s) of assessment do you use? | 0.73 | 0.73 | 2.67 | This item was rated as relevant but needs some revisions and essential. This item was re-run in the second round of content validity. |
| 9b. Which health practitioners do you refer to for the assessment of ocular allergy? | - | - | - | This item was not run as this was a follow-up question. This item was also not re-run. |
| 10a. When managing your ocular allergy patients which method(s) do you use? | 1 | 1 | 2.86 | This item was rated as relevant and essential. Therefore, this item was kept. |
| 10b. Rank these treatments in order, with 1 being the first treatment you recommend… | 0.93 | 0.6 | 2.93 | This item was rated as relevant and essential. Therefore, this item was kept. |
| 11a. Which prevention strategies do you use? | 0.8 | 0.6 | 2.93 | This item was rated as relevant and essential. Therefore, this item was kept. |
| 11b. For which type of ocular allergy do you recommend ‘avoiding allergen triggers’? | 0.73 | 0.06 | 2.93 | This item was rated as relevant but needs some revisions and not essential. This item was removed as it did not add any extra value to the information in the survey. |
| 11c. For which type of ocular allergy do you recommend ‘cease lens wear’? | 0.8 | 0.06 | 2.93 | This item was rated as relevant but not essential. This item was removed as it did not add any extra value to the information in the survey. |
| 11d. For which type of ocular allergy do you recommend to ‘avoid eye rubbing’? | 0.86 | 0.3 | 2.8 | This item was rated as relevant but not essential. This item was removed as it did not add any extra value to the information in the survey. |
| 12a. Which therapeutic strategies do you use? | 0.86 | 0.46 | 2.8 | This item was rated as relevant but not essential. This item was re-run in the second round of content validity. |
| 12bi. For which type of ocular allergy do you recommend ‘cold compresses’? | 0.73 | 0.3 | 3 | This item was rated as relevant but needs some revisions and not essential. This item was removed as it did not add any extra value to the information in the survey. |
| 12bii. What is your prescribing pattern for ‘cold compresses’? | 0.66 | -0.06 | 2.73 | This item was rated as not relevant and not essential. Therefore, this item was removed. |
| 12ci. For which type of ocular allergy do you recommend ‘tear supplements’? | 0.6 | 0.06 | 3 | This item was rated as not relevant and not essential. Therefore, this item was removed. |
| 12cii. What is your prescribing pattern for ‘tear supplements’? | 0.73 | 0.6 | 2.73 | This item was rated as relevant but needs some revisions and essential. This item was removed as it did not add any extra value to the information in the survey. |
| 13a. Which topical allergy eye drops do you recommend? | 0.93 | 0.73 | 2.93 | This item was rated as relevant and essential. Therefore, this item was kept. |
| 13bi. For which type of ocular allergy do you recommend ‘vasoconstrictors’ (e.g. Naphazoline)? | 0.86 | 0.3 | 2.73 | This item was rated as relevant but not essential. This item was re-run in the second round of content validity. |
| 13bii. What is your prescribing pattern for ‘vasoconstrictors’ (e.g. Naphazoline)? | 0.66 | 0.3 | 2.8 | This item was rated as not relevant and not essential. However, this item was re-run in the second round of content validity due to significance to the topic of OA. |
| 13ci. For which type of ocular allergy do you recommend ‘non-steroidal anti-inflammatory’ eye drops? | 0.86 | 0.46 | 2.8 | This item was rated as relevant but not essential. This item was re-run in the second round of content validity. |
| 13cii. What is your prescribing pattern for ‘non-steroidal anti-inflammatory’ eye drops? | 0.8 | 0.46 | 2.73 | This item was rated as relevant but not essential. This item was re-run in the second round of content validity. |
| 13di. For which type of ocular allergy do you recommend ‘antihistamine’ (e.g. Levocabastine) eye drops? | 0.93 | 0.86 | 2.93 | This item was rated as relevant and essential. Therefore, this item was kept. |
| 13dii. What is your prescribing pattern for ‘antihistamine’ (e.g. Levocabastine) eye drops? | 0.86 | 0.6 | 2.73 | This item was rated as relevant and essential. Therefore, this item was kept. However, following content validity round 2, this was removed to maintain consistency. |
| 13ei. For which type of ocular allergy do you recommend ‘mast cell stabiliser’ (e.g. Lodoxamide or Sodium Cromoglycate) eye drops? | 0.86 | 0.73 | 2.93 | This item was rated as relevant and essential. Therefore, this item was kept. |
| 13eii. What is your prescribing pattern for ‘mast cell stabiliser’ (e.g. Lodoxamide or Sodium Cromoglycate) eye drops? | 0.8 | 0.6 | 2.73 | This item was rated as relevant and essential. Therefore, this item was kept. However, following content validity round 2, this was removed to maintain consistency. |
| 13fi. For which type of ocular allergy do you recommend ‘steroid’ eye drops? | 0.86 | 0.73 | 2.86 | This item was rated as relevant and essential. Therefore, this item was kept. |
| 13fii. What is your prescribing pattern for ‘steroid’ eye drops? | 0.8 | 0.6 | 2.73 | This item was rated as relevant and essential. Therefore, this item was kept. However, following content validity round 2, this was removed to maintain consistency. |
| 13gi. For which type of ocular allergy do you recommend an antihistamine-mast cell stabiliser “combination” (e.g. Azelastine, Ketotifen or Olopatadine) eye drop? | 0.86 | 0.73 | 2.93 | This item was rated as relevant and essential. Therefore, this item was kept. |
| 13gii. What is your prescribing pattern for antihistamine-mast cell stabiliser “combination” (e.g. Azelastine, Ketotifen or Olopatadine) eye drop? | 0.8 | 0.6 | 2.73 | This item was rated as relevant and essential. Therefore, this item was kept. However, following content validity round 2, this was removed to maintain consistency. |
| 13hi. For which type of ocular allergy do you recommend an antihistamine-vasoconstrictor “combination” (e.g. Antazoline or Pheniramine) eye drop? | 0.8 | 0.3 | 2.8 | This item was rated as relevant but not essential. This item was re-run in the second round of content validity. |
| 13hii. What is your prescribing pattern for an antihistamine-vasoconstrictor “combination” (e.g. Antazoline or Pheniramine) eye drop? | 0.73 | 0.3 | 2.73 | This item was rated as relevant but needs some revisions and not essential. This item was re-run in the second round of content validity. |
| 13ii. For which type of ocular allergy do you recommend ‘steroid’ eye ointments? | 0.86 | 0.73 | 2.86 | This item was rated as relevant and essential. Therefore, this item was kept. |
| 13iii. What is your prescribing pattern for ‘steroid’ eye ointments? | 0.73 | 0.46 | 2.53 | This item was rated as relevant but needs some revisions and not essential. This item was re-run in the second round of content validity. |
| 14a. Which systemic treatments do you recommend? | 1 | 0.86 | 2.6 | This item was rated as relevant and essential. Therefore, this item was kept. |
| 14bi. For which type of ocular allergy do you recommend ‘oral antihistamines’? | 0.93 | 0.73 | 2.86 | This item was rated as relevant and essential. Therefore, this item was kept. |
| 14bii. What is your prescribing pattern for ‘oral antihistamines’? | 0.8 | 0.6 | 2.67 | This item was rated as relevant and essential. Therefore, this item was kept. However, following content validity round 2, this was removed to maintain consistency. |
| 14ci. For which type of ocular allergy do you recommend ‘nasal antihistamines’? | 1 | 0.6 | 2.93 | This item was rated as relevant and essential. Therefore, this item was kept. |
| 14cii. What is your prescribing pattern for ‘nasal antihistamines’? | 0.8 | 0.46 | 2.73 | This item was rated as relevant but not essential. This item was re-run in the second round of content validity. |
| 14d. For which type of ocular allergy do you recommend ‘allergen specific immunotherapy’? | 1 | 0.86 | 2.8 | This item was rated as relevant and essential. Therefore, this item was kept. |
| 15a. In addition to your other management method(s), which health practitioner(s) do you refer your patients to? | 0.93 | 0.6 | 2.73 | This item was rated as relevant and essential. Therefore, this item was kept. |
| 15b. For which type of ocular allergy do you refer to a ‘general practitioner’ for? | 0.8 | 0.2 | 2.86 | This item was rated as relevant but not essential. This item was removed due to the length of the survey and lack of value in the question. |
| 15c. For which type of ocular allergy do you refer to an ‘optometrist’ for? | 0.73 | 0.3 | 2.86 | This item was rated as relevant but needs some revisions and not essential. This item was removed due to the length of the survey and lack of value in the question. |
| 15d. For which type of ocular allergy do you refer to an ‘ophthalmologist’ for? | 0.8 | 0.6 | 2.93 | This item was rated as relevant and essential. However, this item was removed due to the length of the survey and lack of value in the question. |
| 15e. For which type of ocular allergy do you refer to an ‘allergist’ for? | 0.8 | 0.6 | 2.93 | This item was rated as relevant and essential. However, this item was removed due to the length of the survey and lack of value in the question. |
| 15f. For which type of ocular allergy do you refer to a ‘pharmacist’ for? | 0.6 | -0.3 | 2.73 | This item was rated as not relevant and not essential. Therefore, this item was removed. |
| 15g. For which type of ocular allergy do you refer to ‘other’ specialist for? | 0.53 | -0.2 | 2.53 | This item was rated as not relevant and not essential. Therefore, this item was removed. |
| 16. As you do not manage ocular allergy, which of the following health practitioners do you refer to for your patient to undergo treatment? | 0.93 | 0.73 | 2.86 | This item was rated as relevant and essential. Therefore, this item was kept. |
| 17a. Do you think that some topical anti-allergy eyedrops can aid in eosinophil inhibition? | 0.86 | -0.2 | 2.53 | This item was rated as relevant but not essential. This item was re-run in the second round of content validity. |
| 17b. Do you think that long term use of topical vasoconstrictors can have adverse side effects? | 0.93 | 0.6 | 2.73 | This item was rated as relevant and essential. Therefore, this item was kept. |
| 17c. Do you think that mast cell stabilisers can be used as a prophylactic measure in ocular allergy? | 0.93 | 0.6 | 2.86 | This item was rated as relevant and essential. Therefore, this item was kept. |
| 18a. How often does a patient present to you FIRST (i.e. prior to other health practitioners) for their ocular allergy? | 0.86 | 0.46 | 2.73 | This item was rated as relevant but not essential. However, this item was removed as it was stated it would be hard to ‘elicit accurate information as patients may not always be forthcoming with who they have seen first prior to seeing a given health practitioner’. |
| 18b. Do you think there is a clear collaborative care model between different health practitioners for ocular allergy in Australia? | 0.8 | 0.6 | 2.6 | This item was rated as relevant and essential. Therefore, this item was kept. |
| 18c. What do you think your scope of practice is in diagnosis and treatment of ocular allergy? | 0.66 | -0.06 | 2.33 | This item was rated as not relevant and not essential. Therefore, this item was removed. |
| 19. Do you have any additional information? | - | - | - | This item was not run as it was a general question. |

| Content Validity Round 2 | | | | |
| --- | --- | --- | --- | --- |
| ITEM | **RELEVANCE** | **ESSENTIALITY** | **CLARITY** | **DECISION** |
| 2a. Do you USE any of the following ocular allergy focused quality of life questionnaires in your practice? | - | -0.3 | - | Only essentiality was run in content validity round 2 as this was flagged in round 1. The item was rated as not essential. However, this item was kept but restructured as no other items covered quality of life in the rest of the survey and this topic is considered significant to OA. |
| 2c. Choose the reason why you DO NOT USE these questionnaires. | - | -0.06 | - | Only essentiality was run in content validity round 2 as this was flagged in round 1. The item was rated as not essential. However, this item was kept but restructured as it was related to 2a. |
| 3a. For what purpose do you USE quality of life questionnaires and/or ask quality of life questions. | - | -0.2 | - | Only essentiality was run in content validity round 2 as this was flagged in round 1. The item was rated as not essential. Thus, it was removed as other 2a, 2b, and 2c covered the basis for quality of life. |
| 4b. Choose the reason(s) why you selected “None of the Above/Unsure”. | - | -0.3 | - | Only essentiality was run in content validity round 2 as this was flagged in round 1. This item was rated as not essential. However, this item was kept but slightly reworded as it was related to 4a. |
| 7b. How often do you ask the patient if they rub their eyes? | - | 0.46 | - | Only essentiality was run in content validity round 2 as this was flagged in round 1. This item was rated as not essential. However, this question was kept but slightly reworded due to its significance in OA and no other item covering the basis for this in the survey. |
| 8. Which of these do you consider in your differential diagnosis of ocular allergy? | - | 0.46 | - | Only essentiality was run in content validity round 2 as this was flagged in round 1. This item was rated as not essential. Thus, this item was removed, and the domain removed as this item is covered in the red eye case scenario which was added in round 2. |
| 9a. When diagnosing ocular allergy, which diagnostic method(s) of assessment do you use? | 0.8 | - | - | Only relevance was run in content validity round 2 as this was flagged in round 1. This item was rated as relevant. Thus, it was kept. |
| 12a. Which therapeutic strategies do you use? | - | 0.6 | - | Only essentiality was run in content validity round 2 as this was flagged in round 1. This item was rated as essential. Thus, it was kept. |
| 13bi. For which type of ocular allergy do you recommend ‘vasoconstrictors’ (e.g. Naphazoline)? | - | 0.2 | - | Only essentiality was run in content validity round 2 as this was flagged in round 1. This item was rated as not essential. However, as this was the only item which covers vasoconstrictors in the survey, it was kept. This item was slightly reworded. |
| 13bii. What is your prescribing pattern for ‘vasoconstrictors’ (e.g. Naphazoline)? | 0.8 | 0.2 | - | Both relevance and essentiality were run as they were flagged in round 1. This item was rated as relevant but once again not essential. Therefore, this item was removed. |
| 13ci. For which type of ocular allergy do you recommend ‘non-steroidal anti-inflammatory’ eye drops? | - | 0.3 | - | Only essentiality was run in content validity round 2 as this was flagged in round 1. This item was rated as not essential. However, as this was the only item which covers non-steroidal anti-inflammatory drops in the survey, it was kept. This item was slightly reworded. |
| 13cii. What is your prescribing pattern for ‘non-steroidal anti-inflammatory’ eye drops? | - | 0.06 | - | Only essentiality was run in content validity round 2 as this was flagged in round 1. This item was rated as not essential. Therefore, this item was removed. |
| 13hi. For which type of ocular allergy do you recommend an antihistamine-vasoconstrictor “combination” (e.g. Antazoline or Pheniramine) eye drop? | - | 0.46 | - | Only essentiality was run in content validity round 2 as this was flagged in round 1. This item was rated as not essential. However, as this was the only item which covers antihistamine-vasoconstrictor combination drops in the survey, it was kept. This item was slightly reworded. |
| 13hii. What is your prescribing pattern for an antihistamine-vasoconstrictor “combination” (e.g. Antazoline or Pheniramine) eye drop? | 0.8 | 0.2 | - | Both relevance and essentiality were run as they were flagged in round 1. This item was rated as relevant but once again not essential. Therefore, this item was removed. |
| 13iii. What is your prescribing pattern for ‘steroid’ eye ointments? | 0.8 | 0.6 | - | Both relevance and essentiality were run as they were flagged in round 1. This item was rated as relevant and essential. However, this item was removed to maintain consistency in the survey with other similar questions. Furthermore, steroid ointments was covered in 13ii. |
| 14cii. What is your prescribing pattern for ‘nasal antihistamines’? | - | -0.06 | - | Only essentiality was run in content validity round 2 as this was flagged in round 1. This item was rated as not essential. Therefore, this item was removed. |
| 17a. Do you think that some topical anti-allergy eyedrops can aid in eosinophil inhibition? | - | -0.46 | - | Only essentiality was run in content validity round 2 as this was flagged in round 1. This item was rated as not essential. However, as there was clarity issues in the first round, then this item was kept but restructured. |
| NEW ITEMS | **RELEVANCE** | **ESSENTIALITY** | **CLARITY** | **DECISION** |
| 13biii. How many days do you recommend ‘vasoconstrictors’ (e.g. Naphazoline or Tetryzoline) for? | 0.86 | 0.06 | 2.73 | This item was rated as relevant but not essential. This item was removed. |
| 13fiii. How many days do you recommend ‘steroid’ eye drops for? | 0.93 | 0.73 | 2.73 | This item was rated as relevant and essential. However, this question was removed as the notion that it is on the basis of ‘severity’ was raised throughout the survey. |
| 13iiii. How many days do you recommend ‘steroid’ eye ointments for? | 0.86 | 0.6 | 2.73 | This item was rated as relevant and essential. However, this question was removed as the notion that it is on the basis of ‘severity’ was raised throughout the survey. |
| 17d. Do you think that topical steroid eye drops/ointments can have adverse side effects? | 0.73 | 0.46 | 2.93 | This item was rated as relevant but needs some revisions and not essential. Therefore, this item was kept but restructured. |
| 17e. Are you aware of any side effects of use of topical NSAID eye drops? | 0.86 | 0.3 | 2.86 | This item was rated as relevant but not essential. Therefore, this item was kept but restructured. |
| 13ji. For which type of ocular allergy/for what reason do you recommend ‘other’ eye drops for? | 0.6 | 0.06 | 2.86 | This item was rated as not relevant and not essential. Therefore, this item was removed. |
| 13jii. What is your prescribing pattern for ‘other’ eye drops? | 0.53 | -0.06 | 2.53 | This item was rated as not relevant and not essential. Therefore, this item was removed. |
| 14ei. For which type of ocular allergy/for what reason do you recommend ‘other’ systemic treatments for? | 0.73 | 0.06 | 2.73 | This item was rated as relevant but needs some revisions and not essential. Therefore, this item was removed. |
| 14eii. What is your prescribing pattern for ‘other’ systemic treatments? | 0.73 | -0.2 | 2.73 | This item was rated as relevant but needs some revisions and not essential. Therefore, this item was removed. |
| 18d. Have you ever referred your ocular allergy patient to another health practitioner? | 0.93 | 0.6 | 2.93 | This item was rated as relevant and essential. Therefore, this item was kept. |
| 18e. Which health practitioner do you normally refer to? | 1 | 0.6 | 2.86 | This item was rated as relevant and essential. Therefore, this item was kept. |
| 18f. For what reason do you refer to the above selected practitioner? | 0.93 | 0.3 | 3 | This item was rated as relevant but not essential. However, this was kept do to its significance to the topic of collaborative care and not being covered elsewhere in the survey. |
| 20a. What additional questions would you like to ask? | 0.93 | 0.6 | 3 | This item was rated as relevant and essential (to the clinical case scenario). Therefore, this question was kept. However, this question was restructured as it was initially open ended. It was made as an item with options. |
| 20b. What are your differential diagnoses at this point? | 0.86 | 0.46 | 2.73 | This item was rated as relevant but not essential. However, as differential diagnosis was removed from the rest of the survey, it was kept in the clinical case scenario. |
| 20c. You may or may not choose to diagnose this patient, but what is your suspected diagnosis for this patient? | 0.86 | 0.46 | 2.8 | This item was rated as relevant but not essential. However, it is essential to find a diagnosis in the case scenario and thus, it was kept. |
| 20d. How would you manage this patient? | 0.86 | 0.46 | 2.67 | This item was rated as relevant but not essential. However, it is essential to establish a management in the case scenario and thus, it was kept. |
| 20e. What are your next management steps from here? | 0.86 | 0.46 | 2.86 | This item was rated as relevant but not essential. However, as more information was provided to the participant then it is significant to establish their further management steps. Thus, this item was kept. |
| 20f. Would you refer to another health practitioner? | 0.93 | 0.6 | 3 | This item was rated as relevant and essential. Therefore, this item was kept. |
| 20g. Which health practitioner(s) would you refer to? | 0.86 | 0.6 | 3 | This item was rated as relevant and essential. Therefore, this item was kept. |

| Pilot Study | |  |
| --- | --- | --- |
| ITEM | **DECISION** | **PREVIOUS NUMBER IN CONTENT VALIDITY** |
| 1a. What additional questions would you like to ask? | Kept | 20a |
| 1b. What are your differential diagnosis at this point? | Kept | 20b |
| 1c. You may or may not choose to diagnose this patient, but what is your suspected/potential diagnosis for this patient? | Kept | 20c |
| 1d. How would you manage this patient? | Kept | 20d |
| 1e. What are your next management steps from here? | Kept | 20e |
| 1f. Would you refer to another health practitioner? | Kept | 20f |
| 1g. Which health practitioner(s) would you refer to? | Kept | 20g |
| 2a. Do you ask your ocular allergy patients about their quality of life? | Kept | 2a |
| 2b. How do you ask your ocular allergy patients about their quality of life? | Kept | 2b |
| 2c. Choose the reason(s) why you do not ask your ocular allergy patients about their quality of life? | Kept | 2c |
| 3a. Which types of ocular allergy are you aware of [i.e. aware of their names and how they present]? | Kept | 4a |
| 3b. Choose the reason(s) why you selected “None of the Above/Unsure”. | Kept | 4b |
| 4a. Which of the following is the hallmark symptom of ocular allergy? | Kept | 5 |
| 4b. Which symptom(s) do you ask your patients about when diagnosing ocular allergy? | Kept | 6 |
| 5. How often do you ask your ocular allergy patients if they rub their eyes? | Kept | 7b |
| 6a. When diagnosing ocular allergy, which diagnostic method(s) of assessment do you use? | Kept | 9a |
| 6b. Which health practitioners do you refer to for assessment of ocular allergy? | Kept | 9b |
| 7a. When managing your ocular allergy patients which method(s) do you use? | Kept | 10a |
| 7b. Rank these treatments in order, with 1 being the first treatment you recommend… | Kept | 10b |
| 8. Which prevention strategies do you use? | Kept | 11a |
| 9. Which therapeutic strategies do you use? | Kept | 12a |
| 10a. Which topical allergy eye drops/ointments do you recommend? | Kept | 13a |
| 10b. For which type of ocular allergy/for what reason do you recommend ‘vasoconstrictor (e.g. Naphazoline, or Tetryzoline) eye drops? | Removed | 13bi |
| 10c. For which type of ocular allergy/for what reason do you recommend ‘non-steroidal anti-inflammatory [NSAIDS]’ (e.g. Diclofenac, Ketorolac, or Nepafenac) eye drops? | Removed | 13ci |
| 10d. For which type of ocular allergy/for what reason do you recommend ‘antihistamine’ (e.g. Levocabastine) eye drops? | Removed | 13di |
| 10e. For which type of ocular allergy/for what reason do you recommend ‘mast cell stabiliser’ (e.g. Lodoxamide, or Cromoglycate) eye drops? | Removed | 13ei |
| 10f. For which type of ocular allergy/for what reason do you recommend ‘corticosteroid’ (e.g. Dexamethasone, Fluorometholone, or Prednisolone) eye drops? | Removed | 13fi |
| 10g. For which type of ocular allergy/for what reason do you recommend an ‘antihistamine-mast cell stabiliser combination’ (e.g. Azelastine, Ketotifen, or Olopatadine) eye drops? | Removed | 13gi |
| 10h. For which type of ocular allergy/for what reason do you recommend an ‘antihistamine-vasoconstrictor combination’ (e.g. Antazoline, or Pheniramine) eye drops? | Removed | 13hi |
| 10i. For which type of ocular allergy/for what reason do you recommend ‘corticosteroid’ (e.g. Hydrocortisone) eye ointments? | Removed | 13ii |
| 11a. Which systemic treatments do you recommend? | Kept | 14a |
| 11b. For which types of ocular allergy/for what reason do you recommend ‘oral antihistamines’? | Removed | 14bi |
| 11c. For which type of ocular allergy/for what reason do you recommend ‘nasal antihistamines’? | Removed | 14ci |
| 11d. For which type of ocular allergy/for what reason do you recommend ‘allergen specific immunotherapy’? | Removed | 14d |
| 12a. In addition to you other management method(s), which health practitioner(s) do you refer your ocular allergy patients to? | Kept | 15a |
| 12b. As you do not manage ocular allergy, which of the following health practitioners do you refer to for your patients to undergo treatment? | Kept | 16 |
| 13a. What properties (apart from antihistamine and mast cell control) do some anti-allergy eye drops possess? | Kept | 17a |
| 13b. Which of these are side effects and precautions of vasoconstrictor use? | Kept | 17b |
| 13c. Mast cell stabilisers eye drops are indicated for… | Kept | 17c |
| 13d. Which of these are side effects and precautions of corticosteroid use? | Kept | 17d |
| 13e. Which of the following is responsible for itching in ocular allergy? | Kept | New |
| 13f. Which of these are side effects and precautions of non-steroidal anti-inflammatory [NSAID] use? | Kept | 17e |
| 13gi. Have you recommended calcineurin inhibitors (e.g. cyclosporin, or tacrolimus) for your ocular allergy patients? | Kept | New |
| 13gii. For which type of ocular allergy/for what reason do you recommend ‘calcineurin inhibitors’ (e.g. cyclosporin, or tacrolimus)? | Removed | New |
| 13h. Do you generally prescribe ocular lubricants alongside topical allergy eye drops? | Kept, but restructured. | New |
| 14a. Do you think there is a clear collaborative care model between different health practitioners for ocular allergy in Australia? | Kept | 18b |
| 14b. Have you ever referred your ocular allergy patient to another health practitioner? | Kept | 18d |
| 14c. Which health practitioner(s) do you normally refer to? | Kept | 18e |
| 14d. For what reason do you refer to the above selected health practitioner(s)? | Kept | 18f |
| 15. Do you have any additional information? | Kept | 19 |

| Test-Retest Reliability | | | | | | | |
| --- | --- | --- | --- | --- | --- | --- | --- |
| ITEM | **METHOD OF ANALYSIS** | **PERCENTAGE CHANGE AVERAGE (%)** | **PERCENTAGE CHANGE STANDARD DEVIATION** | **PERCENTAGE AGREEMENT** | **95% CONFIDENCE INTERVAL FOR ICC** | **ICC AVERAGE** | **PERCENTAGE OF FULL AND PARTIAL AGREEMENT** |
| 1a. What additional questions relating to the presenting complaint would you like to ask? | Percentage Change | 4.2857143 | 14.580296 | - | - | - | - |
| 1b. What are your differential diagnoses at this point? | Percentage Change | 0.3809524 | 10.551676 | - | - | - | - |
| 1c. You may or may not choose to diagnose this patient, but what is your suspected/potential diagnosis for this patient? | Percentage Agreement | - | - | 0.8400 | 0.6856-0.9944 | - | - |
| 1d. How would you manage this patient? | Percentage Agreement | - | - | 0.8400 | 0.6856-0.9944 | - | - |
| 1e. What are your next management steps from here? | Percentage Agreement | - | - | 0.8400 | 0.6856-0.9944 | - | - |
| 1f. Would you refer to another health practitioner? | Percentage Agreement | - | - | 0.8400 | 0.6856-0.9944 | - | - |
| 1g. Which health practitioner(s) would you refer to? | Percentage Change | 6 | 16.583124 | - | - | - | - |
| 2a. Do you ask your ocular allergy patients about their quality of life? | Percentage Agreement | - | - | 0.9600 | 0.8774-1.0000 | - | - |
| 2b. How do you ask your ocular allergy patients about their quality of life? | Percentage Change | 0.4444444 | 3.902094 | - | - | - | - |
| 2c. Choose the reason(s) why you do not ask your ocular allergy patients about their quality of life? | Percentage Change | -2.4 | 15.620499 | - | - | - | - |
| 3a. Which types of ocular allergy are you aware of [i.e. aware of their names and how they present]? | Percentage Change | 0.5714286 | 19.552824 | - | - | - | - |
| 3b. Choose the reason(s) why you selected “None of the Above/ Unsure”. | Ignored – Only One Response | - | - | - | - | - | - |
| 4a. Which of the following is the hallmark symptom of ocular allergy? | Percentage Agreement | - | - | 0.7200 | 0.5308-0.9092 | - | - |
| 4b. Which symptom(s) do you ask your patients about when diagnosing ocular allergy? | Percentage Change | 2.4 | 31.73268 | - | - | - | - |
| 5. How often do you ask your ocular allergy patients if they rub their eyes? | Intraclass Correlation Coefficient (ICC) | - | - | - | 0.492372-0.8990608 | 0.7725948 | - |
| 6a. When diagnosing ocular allergy, which diagnostic method(s) of assessment do you use? | Percentage Change | 1.5 | 15.860722 | - | - | - | - |
| 6b. Which health practitioners do you refer to for your additional diagnostic assessment of ocular allergy? | Percentage Change | -3 | 19.525624 | - | - | - | - |
| 7a. When managing your ocular allergy patients which method(s) do you use? | Intraclass Correlation Coefficient (ICC) | - | - | - | 0.6776027-0.968893 | 0.9142212 | - |
| 7b. Order these treatments, with 1 being the first treatment you recommend… | Percentage Agreement | - | - | 0.2400 | - | - | - |
| 8. Which prevention strategies do you recommend? | Percent of Agreement for Dichotomised Index | - | - | - | - | - | 80% |
| 9. Which symptom and cosmetic remedies do you recommend? | Percent of Agreement for Dichotomised Index | - | - | - | - | - | 68% |
| 10a. Which topical allergy eye drops do you recommend? | Percent of Agreement for Dichotomised Index | - | - | - | - | - | 100% |
| 10b. Which topical anti-inflammatory eye drops/ointments do you recommend? | Percent of Agreement for Dichotomised Index | - | - | - | - | - | 88% |
| 11a. Which systemic treatments do you recommend? | Percent of Agreement for Dichotomised Index | - | - | - | - | - | 68% |
| 12a. In addition to your other management method(s), which health practitioner(s) do you refer your ocular allergy patients to? | Percentage Change | 4 | 21.262251 | - | - | - | - |
| 12b. As you do not manage ocular allergy, which of the following health practitioners do you refer to for your patients to undergo treatment? | Ignored – Only One Response | - | - | - | - | - | - |
| 13a. What properties (apart from antihistamine and mast cell control) do some anti-allergy eye drops possess? | Percentage Agreement | - | - | 0.7200 | 0.5308-0.9092 | - | - |
| 13b. Mast cell stabiliser eye drops are most indicated for… | Percentage Agreement | - | - | 0.8000 | 0.6315-0.9685 | - | - |
| 13c. Which of the following is most responsible for itching in ocular allergy? | Percentage Agreement | - | - | 0.8000 | 0.6315-0.9685 | - | - |
| 13d. Which of these are side effects and/or precautions of vasoconstrictor eye drop use, that you consider in your ocular allergy patients? | Percentage Change | 0 | 25.909386 | - | - | - | - |
| 13e. Which of these are side effects and/or precautions of corticosteroid eye drop use, that you consider in your ocular allergy patients? | Percentage Change | 9 | 22.395591 | - | - | - | - |
| 13f. Which of these are side effects and/or precautions of non-steroidal anti-inflammatory [NSAID] eye drop use, that you consider in your ocular allergy patients? | Percentage Change | 12.8 | 25.086517 | - | - | - | - |
| 13g. Have you recommended calcineurin inhibitors (e.g. cyclosporin, or tacrolimus) for your ocular allergy patients? | Percentage Agreement | - | - | 0.9600 | 0.8774-1.0000 | - | - |
| 13h. How often do you consider whether eye drops have preservatives in your ocular allergy patients? | Intraclass Correlation Coefficient (ICC) | - | - | - | 0.8135703-0.9635592 | 0.9174691 | - |
| 13i. How often do you communicate to your ocular allergy patients, not to rub their eyes? | Intraclass Correlation Coefficient (ICC) | - | - | - | 0.8395313-0.9690057 | 0.9295499 | - |
| 14a. Do you think there is a clear collaborative care model between different health practitioners for ocular allergy in Australia? | Percentage Agreement | - | - | 0.7600 | 0.5801-0.9399 | - | - |
| 14b. Have you ever referred your ocular allergy patient to another health practitioner? | Percentage Agreement | - | - | 0.9600 | 0.8774-1.0000 | - | - |
| 14c. Which health practitioner(s) do you normally refer to? | Percentage Change | 1 | 16.894279 | - | - | - | - |
| 14d. For what reason do you refer to the above selected health practitioner(s)? | Percent of Agreement for Dichotomised Index | - | - | - | - | - | 88% |
| 15. Do you have any additional information? | - | - | - | - | - | - | - |
