# Supplementary material for: Development and validation of a health practitioner survey on ocular allergy
Source: Sci Rep. 2024 Apr 30;14:9932. doi: 10.1038/s41598-024-60837-6 (PMC11061311; doi:10.1038/s41598-024-60837-6)
Supplement: Supplementary file 2 — Supplementary Information 2. [file 41598_2024_60837_MOESM2_ESM.docx]

### SURVEY ON OCULAR ALLERGY FOR HEALTH PRACTITIONERS (SOAHP)

DEMOGRAPHICS

What is your age? ____________

What is your gender?

- Male
- Female
- Other

What is your main occupation?

- Allergist
- General Practitioner
- Ophthalmologist
- Optometrist
- Pharmacist

Display This Question:

If Occupation = Optometrist

Do you have a therapeutic endorsement?

- Yes
- No

How many years have you been practicing, excluding years of training (round to the nearest whole number)? ____________

What is your main practice postcode (enter ONLY one)? ____________

Do you mainly practice at a...

- Solo or Individual Practice (i.e. you are the only one in this practice)
- Group Practice (i.e. other practicing with you)
- Hospital
- Community Health Centre
- Other. Please Specify: ____________

What is your modality of practice?

- Full-Time
- Part-Time
- Locum or Casual
- Other. Please Specify: ____________

How often do you see ocular allergy patients?

- Daily
- Weekly
- Monthly
- Occasionally (i.e. every 6-12 months)
- Rarely (i.e. once a year)
- Never

How confident are you with your knowledge on ocular allergy?

- Low Confidence
- Medium Confidence
- High Confidence

How regularly do you receive education (e.g. conferences, CPD, articles etc.) surrounding ocular allergy?

- Daily
- Weekly
- Monthly
- Occasionally (i.e. every 6-12 months)
- Rarely (i.e. once a year)
- Never

RED EYE CASE SCENARIO

A 50-year-old gentleman presents to you complaining of redness, itchiness, and burning in his eyes and eyelids. He says it is in both his eyes and started about 6 hours ago after he awoke.

 On observation you can see that both his eyes are red, and the eyelids are also red and swollen. On further questioning you find that he began using a new face cream last night before going to bed. He says that he has not tried to treat this apart from washing his face with water which has provided very mild, temporary relief. However, now that it has not gone away, he has come straight to you.

1a What additional questions relating to the presenting complaint would you like to ask?

- Flashes and Floaters
- Double Vision
- Pain
- Vision (Distance and Near)
- Photophobia (Light Sensitivity)
- Mucous Secretions/Discharge (i.e. Watery, Purulent, Clear)
- Ocular Injury/Surgery/Infections
- Headaches
- Current Medications (e.g. Oral, Eyedrops etc.)
- Allergic History (e.g. Atopy, Food, Drugs etc.)
- Systemic Symptoms (e.g. sneezing, coughing etc.)
- Contact Lens Wear
- Career/Line of Work
- Current Spectacle Wear
- Other. Please Specify: ____________
- Additional Comments: ____________

1b What are your differential diagnoses at this point (select ALL that apply)?

- Acute Allergic Conjunctivitis
- Bacterial Conjunctivitis (Acute, Hyperacute and/or Chronic)
- Seasonal Allergic Conjunctivitis
- Dry Eye (incl. Meibomian Gland Dysfunction and/or Blepharitis)
- Perennial Allergic Conjunctivitis
- Chlamydial Diseases (Adult Inclusion Conjunctivitis and/or Trachoma)
- Vernal Keratoconjunctivitis
- Viral Disease (Viral Conjunctivitis and/or Herpes Simplex Keratitis)
- Atopic Keratoconjunctivitis
- Episcleritis
- Scleritis
- Giant Papillary Conjunctivitis
- Acute Anterior Uveitis
- Contact Blepharoconjunctivitis
- Acute Angle Closure
- Subconjunctival Haemorrhage
- Contact Lens Related Issues (Contact Lens Acute Red Eye, and/or Conjunctival and Limbal Redness)
- Welders Flash/Arc Eye
- Foreign Body
- Microbial Keratitis
- Ocular Ulcer
- None of the Above
- I am unsure
- Other. Please Specify: ____________
- Additional Comments: ____________

The additional information you learn is that:

- He is a builder who works in a dusty environment and does not regularly wear his safety glasses.
- There has been occasional white mucous secretions from both his eyes.
- He also regularly takes oral antihistamines during allergy seasons but is currently not taking any.
- He also recalls an allergic reaction to Chloramphenicol eye drops that he was prescribed from his general practitioner a few years back.
- He does not wear contact lenses and only wears spectacles for reading.
- He has not had any ocular injuries/surgeries/infections.
- He has no other outstanding health conditions, no systemic symptoms, and is on no medications.
- He is experiencing NO blurry vision/double vision/pain/headaches/yellow mucous secretions/flashes/floaters/light sensitivity.

You proceed to perform the appropriate examinations in your practice. All results appear normal apart from what you already observed with the naked eye which is the red conjunctiva in both eyes, and the red, swollen eyelids in both eyes.

1c You may or may not choose to diagnose this patient, but what is your suspected/potential diagnosis for this patient?

- Acute Allergic Conjunctivitis
- Bacterial Conjunctivitis (Acute, Hyperacute and/or Chronic)
- Seasonal Allergic Conjunctivitis
- Dry Eye (incl. Meibomian Gland Dysfunction and/or Blepharitis)
- Perennial Allergic Conjunctivitis
- Chlamydial Diseases (Adult Inclusion Conjunctivitis and/or Trachoma)
- Vernal Keratoconjunctivitis
- Viral Disease (Viral Conjunctivitis and/or Herpes Simplex Keratitis)
- Atopic Keratoconjunctivitis
- Episcleritis
- Scleritis
- Giant Papillary Conjunctivitis
- Acute Anterior Uveitis
- Contact Blepharoconjunctivitis
- Acute Angle Closure
- Subconjunctival Haemorrhage
- Contact Lens Related Issues (Contact Lens Acute Red Eye, and/or Conjunctival and Limbal Redness)
- Welders Flash/Arc Eye
- Foreign Body
- Microbial Keratitis
- Ocular Ulcer
- None of the Above
- I am unsure
- Other. Please Specify: ____________

Whilst typing your notes, he remembers that when he noticed the white mucous secretions he started using ocular lubricants to "wash them out" which only "worsened" the situation. He cannot recall the exact drop but says it's the "regular" one.

1d How would you manage this patient (select ALL that apply)?

- Cold Compresses
- Avoid New Cream Use
- Avoid Eye Rubbing
- Antibacterial Eye Drops/Ointments. Please Specify: ____________
- Tear Supplements/Lubricants. Please Specify: ____________
- Glaucoma Eye Drops. Please Specify: ____________
- Antiviral Eye Drops. Please Specify: ____________
- Vasoconstrictor Eye Drops. Please Specify: ____________
- Non-Steroidal Anti-Inflammatory Eye Drops (NSAIDS). Please Specify: ______
- Antihistamine Eye Drops. Please Specify: ____________
- Mast Cell Stabiliser Eye Drops. Please Specify: ____________
- Antihistamine-Mast Cell Stabiliser Combination Eye Drops. Please Specify: __
- Antihistamine-Vasoconstrictor Combination Eye Drops. Please Specify: _____
- Steroid Eye Drops. Please Specify: ____________
- Steroid Eye Ointments. Please Specify: ____________
- Oral Antihistamines. Please Specify: ____________
- Nasal Antihistamines. Please Specify: ____________
- Allergen Specific Immunotherapy. Please Specify: ____________
- Additional Referral. To whom: ____________
- Follow Up. When: ____________
- I won't manage. I will only refer. To whom: ____________
- I am unsure
- Other. Please Specify: ____________
- Additional Comments: ____________

Skip To: End of Domain If 1d = I won't manage. I will only refer. To whom:

You choose to manage this patient conservatively by advising him to stop using the new face cream, avoid eye rubbing, and use cold compresses. Further to this, you recommend using the oral antihistamines he regularly uses, and you also prescribe him a preservative-free antihistamine/mast cell stabiliser eye drop and a preservative-free lubricant eye drop.

He returns to you 48 hours later reporting that although the symptoms have slightly improved, they are still extremely bothersome. Your clinical examination shows that the signs are also showing very slight improvement. He says he has followed your management plan very closely and you trust he is compliant. Additionally, he has been wearing his safety spectacles at work so as not to worsen his condition. However, he has chosen not to go to work today so that he can see you.

1e What are your next management steps from here (select ALL that apply)?

- Continue Avoiding New Cream Use
- Continue Avoiding Eye Rubbing
- Continue Cold Compresses
- Continue Oral Antihistamines
- Continue Preservative-Free Tear Supplements/Lubricants
- Continue Preservative-Free Antihistamine-Mast Cell Stabiliser Combination Eye Drops
- Antibacterial Eye Drops/Ointments. Please Specify: ____________
- Vasoconstrictor Eye Drops. Please Specify: ____________
- Non-Steroidal Anti-Inflammatory Eye Drops (NSAIDS). Please Specify: ______
- Antihistamine Eye Drops. Please Specify: ____________
- Mast Cell Stabiliser Eye Drops. Please Specify: ____________
- Antiviral Eye Drops. Please Specify: ____________
- Antihistamine-Vasoconstrictor Combination Eye Drops. Please Specify: _____
- Steroid Eye Drops. Please Specify: ____________
- Steroid Eye Ointments. Please Specify: ____________
- Glaucoma Eye Drops. Please Specify: ____________
- Nasal Antihistamines. Please Specify: ____________
- Allergen Specific Immunotherapy. Please Specify: ____________
- Referral. To whom: ____________
- Follow Up. When: ____________
- Other. Please Specify: ____________
- Additional Comments: ____________

Skip To: End of Domain If 1e = Referral. To whom:

1f Would you refer to another health practitioner?
_Please note: Sending patient to the pharmacist for prescription is also considered a referral in this case._

- Yes
- No

Display This Question:

If 1f = Yes

1g Which health practitioner(s) would you refer to (select ALL that apply)?

- General Practitioner
- Optometrist
- Ophthalmologist
- Pharmacist
- Allergist
- Other. Please Specify: ____________
- Additional Comments: ____________

QUALITY OF LIFE IN OCULAR ALLERGY PATIENTS

2a Do you ask your ocular allergy patients about their quality of life?

- Yes
- No

Display This Question:

If 2a = Yes

2b How do you ask your ocular allergy patients about their quality of life (select ALL that apply)?

- I ask my own quality of life questions.
- Rhinoconjunctivitis Quality of Life Questionnaire (RQLQ)
- Standardised Rhinoconjunctivitis Quality of Life Questionnaire (RQLQ(S))
- Mini Rhinoconjunctivitis Quality of Life Questionnaire (MiniRQLQ)
- Nocturnal Rhinoconjunctivitis Quality of Life Questionnaire ((N)RQLQ)
- Adolescent Rhinoconjunctivitis Quality of Life Questionnaire (AdolRQLQ)
- Paediatric Rhinoconjunctivitis Quality of Life Questionnaire (PRQLQ)
- Eye Allergy Patient Impact Questionnaire (EAPIQ)
- Quality of Life of Children with Allergic Keratoconjunctivitis Questionnaire (QUICK)
- Other. Please Specify: ____________
- Additional Comments: ____________

Display This Question:

If 2a = No

2c Choose the reason(s) why you do not ask your ocular allergy patients about their quality of life (select ALL that apply).

- I am not aware of current questionnaires to use.
- It is time consuming.
- Asking about quality of life in ocular allergy patients is not essential.
- Quality of life questions is not part of my normal clinical routine.
- Quality of life will not affect my diagnosis and management of ocular allergy.
- Other. Please Specify: ____________
- Additional Comments: ____________

OCULAR ALLERGY HISTORY QUESTIONS

3a Which types of ocular allergy are you aware of [i.e. aware of their names and how they present] (select ALL that apply)?

- Acute Allergic Conjunctivitis (AAC)
- Seasonal Allergic Conjunctivitis (SAC)
- Perennial Allergic Conjunctivitis (PAC)
- Vernal Keratoconjunctivitis (VKC)
- Atopic Keratoconjunctivitis (AKC)
- Giant Papillary Conjunctivitis (GPC)
- Contact Blepharoconjunctivitis (CBC)
- None of the Above/Unsure
- Other. Please Specify: ____________
- Additional Comments: ____________

Display This Question:

If 3a = None of the Above/Unsure

3b Choose the reason(s) why you selected "None of the Above/Unsure" (select ALL that apply).

- I refer to ocular allergy simply as allergic conjunctivitis.
- I only consider ocular allergy when it is associated with allergic rhinitis (hayfever) and other allergic conditions.
- I am unaware there were different types of ocular allergy.
- I rarely diagnose ocular allergy.
- I mainly diagnose allergic rhinitis (hayfever) and other allergic conditions, then depending on the symptoms I manage accordingly.
- I do not differentiate the types of ocular allergy. I just manage based on severity.
- Other. Please Specify: ____________
- Additional Comments: ____________

4a Which of the following is the hallmark symptom of ocular allergy?

- Eye Rubbing
- Red Eyes
- Burning/Stinging
- Itchy Eyes
- Eyelid Swelling
- Watery Secretions
- White-Stringy Mucous Secretions
- Photophobia (Light Sensitivity)
- Ocular Pain
- Foreign Body Sensation
- Intolerance to Contact Lens Wear
- Blurry Vision
- Unsure

4b Which symptom(s) do you ask your patients about when diagnosing ocular allergy (select ALL that apply)?

- Itchy Eyes
- Eye Rubbing
- Red Eyes
- Burning/Stinging
- Eyelid Swelling
- Watery Secretions
- White-Stringy Mucous Secretions
- Photophobia (Light Sensitivity)
- Ocular Pain
- Foreign Body Sensation
- Intolerance to Contact Lens Wear
- Blurry Vision
- Double Vision
- Allergic History (e.g. Atopy, Food, Drugs etc.)
- Purulent Discharge
- I don't diagnose ocular allergy
- Other. Please Specify: ____________
- Additional Comments: ____________

5 How often do you ask your ocular allergy patients if they rub their eyes?

- Always
- Frequently
- Sometimes
- Rarely
- Never

DIAGNOSTIC METHODS IN OCULAR ALLERGY

6a When diagnosing ocular allergy, which diagnostic method(s) of assessment do you use (select ALL that apply)?

- Slit Lamp Assessment (for Signs of Ocular Allergy)
- Slit Lamp Assessment (for Signs of Differential Diagnosis)
- Tear Film Assessment (Using Slit Lamp)
- Tear Film Assessment (Using Keratography and Other Similar Equipment)
- Topography (Signs of Keratoconus)
- Skin Prick Test
- Serum IgE (i.e. IgE Blood Test)
- I also refer to other health care practitioners to undergo additional assessment
- I do not use diagnostic methods to diagnose ocular allergy. I only consider symptoms when diagnosing ocular allergy patients.
- I do not use diagnostic methods to diagnose ocular allergy. I only refer to other health care practitioners to undergo assessment.
- None of the Above
- Other. Please Specify: ____________
- Additional Comments: ____________

Display This Question:

If 6a = I do not use diagnostic methods to diagnose ocular allergy. I only refer to other health care practitioners to undergo assessment.

Or 6a = I also refer to other health care practitioners to undergo additional assessment

6b Which health practitioners do you refer to for additional diagnostic assessment of ocular allergy (select ALL that apply)?

- General Practitioner
- Optometrist
- Ophthalmologist
- Allergist
- Pharmacist
- Other. Please Specify: ____________
- Additional Comments: ____________

MANAGEMENT METHODS IN OCULAR ALLERGY

7a When managing your ocular allergy patients which method(s) do you use (select ALL that apply)?

- Prevention Strategies (i.e. Avoiding Allergen Triggers, Avoid Eye Rubbing)
- Symptom and Cosmetic Remedies (i.e. Cold Compresses, Ocular Lubricants, Vasoconstrictors)
- Topical Allergy Eye Drops (i.e. Antihistamines, Mast Cell Stabilisers, Combination Drops)
- Topical Anti-Inflammatory Eye Drops/Ointments (i.e. Non-Steroidal Anti-Inflammatories [NSAIDS], Corticosteroids)
- Systemic Treatments (i.e. Oral Antihistamines, Nasal Antihistamines, Allergen Specific Immunotherapy)
- Additional Referral (i.e. General Practitioner, Optometrist, Ophthalmologist, Allergist, Pharmacist, Other)
- I don't usually manage ocular allergy patients. I refer to other health care practitioners to undergo treatment.
- Other. Please Specify: ____________
- Additional Comments: ____________

Display This Question:

If 7a = Prevention Strategies (i.e. Avoiding Allergen Triggers, Avoid Eye Rubbing)

Or 7a = Symptom and Cosmetic Remedies (i.e. Cold Compresses, Ocular Lubricants, Vasoconstrictors)

Or 7a = Topical Allergy Eye Drops (i.e. Antihistamines, Mast Cell Stabilisers, Combination Drops)

Or 7a = Systemic Treatments (i.e. Oral Antihistamines, Nasal Antihistamines, Allergen Specific Immunotherapy)

Or 7a = Additional Referral (i.e. General Practitioner, Optometrist, Ophthalmologist, Allergist, Pharmacist, Other)

Or 7a = Other. Please Specify:

Or 7a = Topical Anti-Inflammatory Eye Drops/Ointments (i.e. Non-Steroidal Anti-Inflammatories [NSAIDS], Corticosteroids)

| 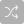 |
| --- |

7b Order these treatments, with 1 being the first treatment you recommend...

Display This Choice:

If 7a = Prevention Strategies (i.e. Avoiding Allergen Triggers, Avoid Eye Rubbing)

______ Prevention Strategies (i.e. Avoiding Allergen Triggers, Avoid Eye Rubbing)

Display This Choice:

If 7a = Symptom and Cosmetic Remedies (i.e. Cold Compresses, Ocular Lubricants, Vasoconstrictors)

______ Symptom and Cosmetic Remedies (i.e. Cold Compresses, Ocular Lubricants, Vasoconstrictors)

Display This Choice:

If 7a = Topical Allergy Eye Drops (i.e. Antihistamines, Mast Cell Stabilisers, Combination Drops)

______ Topical Allergy Eye Drops (i.e. Antihistamines, Mast Cell Stabilisers, Combination Drops)

Display This Choice:

If 7a = Topical Anti-Inflammatory Eye Drops/Ointments (i.e. Non-Steroidal Anti-Inflammatories [NSAIDS], Corticosteroids)

______ Topical Anti-Inflammatory Eye Drops/Ointments (i.e. Non-Steroidal Anti-Inflammatories [NSAIDS], Corticosteroids)

Display This Choice:

If 7a = Systemic Treatments (i.e. Oral Antihistamines, Nasal Antihistamines, Allergen Specific Immunotherapy)

______ Systemic Treatments (i.e. Oral Antihistamines, Nasal Antihistamines, Allergen Specific Immunotherapy)

Display This Choice:

If 7a = Additional Referral (i.e. General Practitioner, Optometrist, Ophthalmologist, Allergist, Pharmacist, Other)

______ Additional Referral (i.e. General Practitioner, Optometrist, Ophthalmologist, Allergist, Pharmacist, Other)

Display This Choice:

If 7a = Other. Please Specify:

______ Other

Display This Question:

If 7a = Prevention Strategies (i.e. Avoiding Allergen Triggers, Avoid Eye Rubbing)

8 Which prevention strategies do you recommend (select ALL that apply)?

- Avoiding Allergen Triggers
- Avoid Eye Rubbing
- Other. Please Specify: ____________
- Additional Comments: ____________

Display This Question:

If 7a = Symptom and Cosmetic Remedies (i.e. Cold Compresses, Ocular Lubricants, Vasoconstrictors)

9 Which symptom and cosmetic remedies do you recommend (select ALL that apply)?

- Cold Compresses
- Ocular Lubricants. Please Specify: ____________
- Vasoconstrictor Eye Drops (e.g. Naphazoline, or Tetryzoline). Please Specify: _
- Other. Please Specify: ____________
- Additional Comments: ____________

Display This Question:

If 7a = Topical Allergy Eye Drops (i.e. Antihistamines, Mast Cell Stabilisers, Combination Drops)

10a Which topical allergy eye drops do you recommend (select ALL that apply)?

- Antihistamine Eye Drops (e.g. Levocabastine). Please Specify: ____________
- Mast Cell Stabiliser Eye Drops (e.g. Lodoxamide, or Cromoglycate). Please Specify: ____________
- Antihistamine-Mast Cell Stabiliser Combination Eye Drops (e.g. Azelastine, Ketotifen, or Olopatadine). Please Specify: ____________
- Antihistamine-Vasoconstrictor Combination Eye Drops (e.g. Antazoline, or Pheniramine). Please Specify: ____________
- Other. Please Specify: ____________
- Additional Comments: ____________

Display This Question:

If 7a = Topical Anti-Inflammatory Eye Drops/Ointments (i.e. Non-Steroidal Anti-Inflammatories [NSAIDS], Corticosteroids)

10b Which topical anti-inflammatory eye drops/ointments do you recommend (select ALL that apply)?

- Non-Steroidal Anti-Inflammatory Eye Drops [NSAIDS] (e.g. Diclofenac, Ketorolac, or Nepafenac). Please Specify: ____________
- Corticosteroid Eye Drops (e.g. Dexamethasone, Fluorometholone, or Prednisolone). Please Specify: ____________
- Corticosteroid Eye Ointments (e.g. Hydrocortisone). Please Specify: ____________
- Other. Please Specify: ____________
- Additional Comments: ____________

Display This Question:

If 7a = Systemic Treatments (i.e. Oral Antihistamines, Nasal Antihistamines, Allergen Specific Immunotherapy)

11 Which systemic treatments do you recommend (select ALL that apply)?

- Oral Antihistamines
- Nasal Antihistamines
- Allergen Specific Immunotherapy
- Other. Please Specify: ____________
- Additional Comments: ____________

Display This Question:

If 7a = Additional Referral (i.e. General Practitioner, Optometrist, Ophthalmologist, Allergist, Pharmacist, Other)

12a In addition to your other management method(s), which health practitioner(s) do you refer your ocular allergy patients to (select ALL that apply)?
_Please note: Sending patient to the pharmacist for prescription is also considered a referral in this case._

- General Practitioner
- Optometrist
- Ophthalmologst
- Allergist
- Pharmacist
- Other. Please Specify: ____________
- Additional Comments: ____________

Display This Question:

If 7a = I don't usually manage ocular allergy patients. I refer to other health care practitioners to undergo treatment.

12b As you do not manage ocular allergy, which of the following health practitioners do you refer to for your patients to undergo treatment (select ALL that apply)? 
_Please note: Sending patient to the pharmacist for prescription is also considered a referral in this case._

- General Practitioner
- Optometrist
- Ophthalmologist
- Allergist
- Pharmacist
- Other. Please Specify: ____________
- Additional Comments: ____________

KNOWLEDGE ON OCULAR ALLERGY

_Please answer the following questions based on your current knowledge for the purpose of this research (i.e. without using google etc.). Your responses will not be revealed to anyone beyond the researchers in this study._

13a What properties (apart from antihistamine and mast cell control) do some anti-allergy eye drops possess (select the most CORRECT response)?

- Neutrophil Inhibition
- Eosinophil Inhibition
- Basophil Inhibition
- Unsure
- Other. Please Specify: ____________

13b Mast cell stabilisers eye drops are most indicated for...

- Reduction of Red Eyes (i.e. constriction of vessels in conjunctiva)
- Prophylaxis (i.e. using before allergy season)
- Antibacterial Properties (i.e. prevent infections with ocular allergy)
- Unsure
- Other. Please Specify: ____________

13c Which of the following is most responsible for itching in ocular allergy?

- IgE Antibodies
- Antigen Presenting Cells
- Histamine
- T Cells and B Cells
- Unsure
- Other. Please Specify: ____________

13d Which of these are side effects and/or precautions of vasoconstrictor eye drop use, that you consider in your ocular allergy patients (select ALL that apply)?

- Mydriasis (i.e. Pupil Dilation)
- Glaucoma (e.g. Acute Angle Closure Glaucoma)
- Rebound Hyperaemia
- Eye Infections
- Stinging on Instillation
- Blurred Vision
- Unsure
- None of the Above
- Other. Please Specify: ____________
- Additional Comments: ____________

13e Which of these are side effects and/or precautions of corticosteroid eye drop use, that you consider in your ocular allergy patients (select ALL that apply)?

- Risk of Infection
- Increased Intraocular Pressure
- Cataracts (e.g. Posterior Subcapsular Cataract)
- Photophobia (i.e. Light Sensitivity)
- Blurred Vision
- Mydriasis (i.e. Pupil Dilation)
- Delayed Wound Healing
- Rebound Inflammation
- Unsure
- None of the Above
- Other. Please Specify: ____________
- Additional Comments: ____________

13f Which of these are side effects and/or precautions of non-steroidal anti inflammatory [NSAID] eye drop use, that you consider in your ocular allergy patients (select ALL that apply)?

- Delayed Wound Healing
- Stinging/Irritation on Instillation
- Corneal Effects (e.g. corneal ulceration, keratitis etc.)
- Masking of Infection
- Use alongside steroids, aspirin, other NSAIDS, prostaglandin analogues etc.
- Unsure
- None of the Above
- Other. Please Specify: ____________
- Additional Comments: ____________

13g Have you recommended calcineurin inhibitors (e.g. cyclosporin, or tacrolimus) for your ocular allergy patients?

- Yes. For what reason: ____________
- No. For what reason: ____________

13h How often do you consider whether eye drops have preservatives in your ocular allergy patients?

- Always
- Frequently
- Sometimes
- Rarely
- Never

13i How often do you communicate to your ocular allergy patients, not to rub their eyes?

- Always
- Frequently
- Sometimes
- Rarely
- Never

COLLABORATIVE CARE IN OCULAR ALLERGY

14a Do you think there is a clear collaborative care model between different health practitioners for ocular allergy in Australia?

- Yes
- No
- Unsure

14b Have you ever referred your ocular allergy patient to another health practitioner?
 Please note: Sending patient to the pharmacist for prescription is also considered a referral in this case.

- Yes
- No

Display This Question:

If 14b = Yes

14c Which health practitioner(s) do you normally refer to (select ALL that apply)?

- General Practitioner
- Optometrist
- Ophthalmologist
- Pharmacist
- Allergist
- Other. Please Specify: ____________
- Additional Comments: ____________

Display This Question:

If 14b = Yes

14d For what reason do you refer to the above selected health practitioner(s) (select ALL that apply)?

- Diagnosis
- Treatment
- Other. Please Specify: ____________
- Additional Comments: ____________

15 Do you have any additional information?

________________________________________________________________

________________________________________________________________

________________________________________________________________

________________________________________________________________

________________________________________________________________
